# Ghrelin in Focus: Dissecting Its Critical Roles in Gastrointestinal Pathologies and Therapies

**DOI:** 10.3390/cimb46010061

**Published:** 2024-01-22

**Authors:** Wei Wu, Lei Zhu, Zhimin Dou, Qiliang Hou, Sen Wang, Ziqian Yuan, Bin Li

**Affiliations:** 1 Department of Intensive Care Medicine, The First School of Clinical Medicine, Lanzhou University, Lanzhou 730000, China; wuw21@lzu.edu.cn (W.W.); qilianghou@outlook.com (Q.H.); 220220909171@lzu.edu.cn (S.W.); yuanzq331@163.com (Z.Y.); 2 Department of Intensive Care Medicine, The First Hospital of Lanzhou University, Lanzhou 730000, China; zhulei0224@163.com (L.Z.); douzhm@126.com (Z.D.)

**Keywords:** ghrelin, gastrointestinal tract, sepsis, inflammatory bowel disease, gastric cancer, colorectal cancer, surgery

## Abstract

This review elucidates the critical role of ghrelin, a peptide hormone mainly synthesized in the stomach in various gastrointestinal (GI) diseases. Ghrelin participates in diverse biological functions ranging from appetite regulation to impacting autophagy and apoptosis. In sepsis, it reduces intestinal barrier damage by inhibiting inflammatory responses, enhancing GI blood flow, and modulating cellular processes like autophagy and apoptosis. Notably, in inflammatory bowel disease (IBD), serum ghrelin levels serve as markers for distinguishing between active and remission phases, underscoring its potential in IBD treatment. In gastric cancer, ghrelin acts as an early risk marker, and due to its significant role in increasing the proliferation and migration of gastric cancer cells, the ghrelin–GHS-R axis is poised to become a target for gastric cancer treatment. The role of ghrelin in colorectal cancer (CRC) remains controversial; however, ghrelin analogs have demonstrated substantial benefits in treating cachexia associated with CRC, highlighting the therapeutic potential of ghrelin. Nonetheless, the complex interplay between ghrelin’s protective and potential tumorigenic effects necessitates a cautious approach to its therapeutic application. In post-GI surgery scenarios, ghrelin and its analogs could be instrumental in enhancing recovery and reducing complications. This article accentuates ghrelin’s multifunctionality, shedding light on its influence on disease mechanisms, including inflammatory responses and cancer progression, and examines its therapeutic potential in GI surgeries and disorders, advocating for continued research in this evolving field.

## 1. Introduction

Ghrelin plays a pivotal role in regulating various gastrointestinal (GI) functions and has emerged as a significant factor in the context of GI diseases. Stimulation of the ghrelin/GHS-R1a pathway influences appetite stimulation, gastric acid and digestive enzyme secretion, GI motility, and energy balance [1]. Recent research has unveiled further functions of ghrelin in the development of various GI diseases, offering potential therapeutic opportunities for intervention.

This review aims to meticulously synthesize the latest scientific findings regarding the role of ghrelin in GI diseases. Through an in-depth analysis of the existing literature, we aim to enhance our understanding of the intricate mechanistic interplay between ghrelin and GI diseases. Additionally, by identifying potential therapeutic strategies, this review may contribute to the development of innovative interventions for the management and treatment of GI diseases. It is anticipated that this synthesis of current research will not only shed light on the intricate functions of ghrelin but also offer potential directions for future investigations and interventions.

This review is structured into three distinct sections to facilitate a narrative analysis. The first section, “Ghrelin’s Impact on the GI System”, elucidates the physiological functions of ghrelin within the GI tract, focusing on its impact on appetite regulation, gastric acid secretion, and GI motility. The second section, “The Mechanisms Underlying the Therapeutic Effects of Ghrelin in GI Diseases”, delves into the molecular mechanisms by which ghrelin influences GI disease pathogenesis and further discusses potential therapeutic applications. Lastly, the third section, “Ghrelin and GI Disorders”, provides an in-depth examination of ghrelin’s involvement in specific GI diseases.

## 2. Ghrelin’s Impact on the GI System

### 2.1. Synthesis of Ghrelin

Ghrelin, a multifunctional GI acylated peptide, plays a pivotal role in modulating diverse biological, physiological, and pathological functions in vertebrates [2]. While the stomach serves as the primary site for ghrelin production, other organs, including the duodenum, hypothalamus, lungs, gonads, and pancreas, also produce small amounts in humans [1]. At the cellular level, P/D1 cells primarily produce ghrelin in humans, while in rats, X/a-like cells are responsible for its synthesis [3]. Ghrelin-producing cells are primarily classified into two distinct categories: closed-type cells, which lack continuity with the GI tract lumen, and open-type cells, which maintain direct contact with the GI tract lumen [4]. Notably, the stomach contains exclusively closed-type cells, whereas the small intestine and the colorectal feature a coexistence of both closed-type and open-type cells [5]. The functional regulation of open-type cells is predominantly guided by chemoreceptors responsive to nutrients. Conversely, closed-type cells, in addition to being regulated by chemoreceptors, are also influenced by a range of other hormones secreted within the GI tract, such as somatostatin and glucagon, reflecting a complex regulatory network within the GI hormonal landscape [6,7].

**Figure 1:**
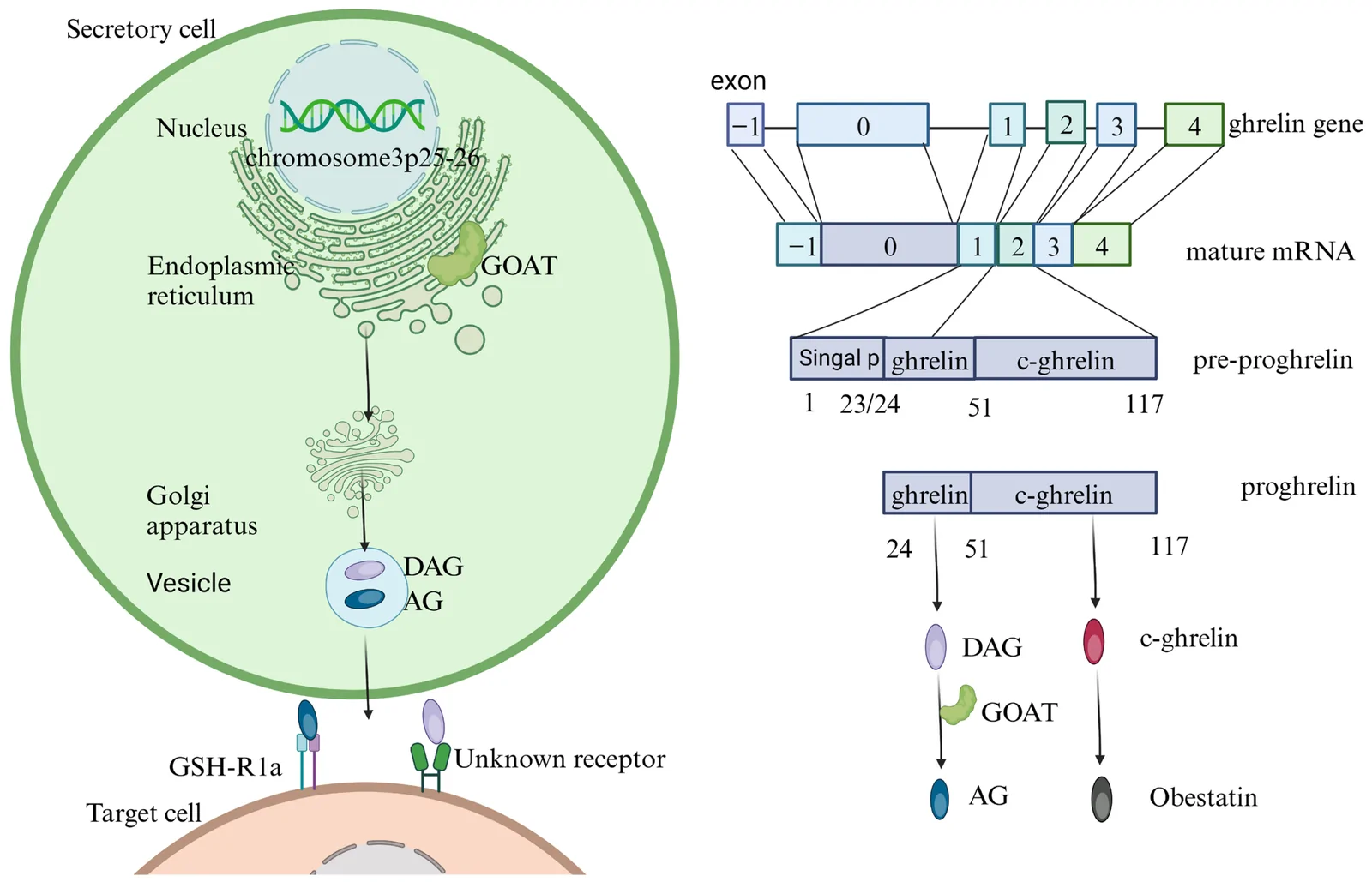
Diagram of ghrelin production and function. The human GHRL gene is located on the short arm of chromosome 3p25-26 and comprises six exons. The ghrelin gene undergoes transcription and splicing to form mature mRNA. This mRNA is translated into pre-proghrelin, a precursor containing 117 amino acids. Within the endoplasmic reticulum, this precursor undergoes a systematic cleavage process, initially forming proghrelin and subsequently being converted into ghrelin, which is then modified by ghrelin O-acyltransferase (GOAT) to form des-acyl ghrelin (DAG) and acyl ghrelin (AG). These forms are subsequently packaged into vesicles in the Golgi apparatus and secreted. AG binds to the growth hormone secretagogue receptor 1a (GHS-R1a) to produce various physiological effects. The receptor with which DAG interacts remains unknown. Signal P: signal peptide.

The synthesis of ghrelin involves several steps (Figure 1). The human ghrelin gene, located on chromosome 3 (3p25-26) [8], spans 7.2 kb and encompasses six exons. It is transcribed and translated into a precursor protein called pre-proghrelin [9]. In the endoplasmic reticulum, proteases cleave pre-proghrelin to produce a ninety-four amino-acid intermediate known as proghrelin [9]. The final step involves the modification of proghrelin by ghrelin-O-acyltransferase (GOAT) to produce mature ghrelin, which is twenty-eight amino acids in length [8]. Ghrelin exists in two forms: acylated ghrelin (AG) and des-acyl ghrelin (DAG). AG is acylated with an octanoyl group on the third amino acid and binds to GHS-R1a, through which it exerts various physiological effects. The function of GHS-R1a is modulated by the relative expression level of GHS-R1b, a truncated nonfunctional receptor [10,11]. DAG lacks acylation and does not bind to GHS-R1a [12]; the receptor that recognizes DAG remains unidentified.

### 2.2. Physiological Effects of Ghrelin on the GI System

**Figure 2:**
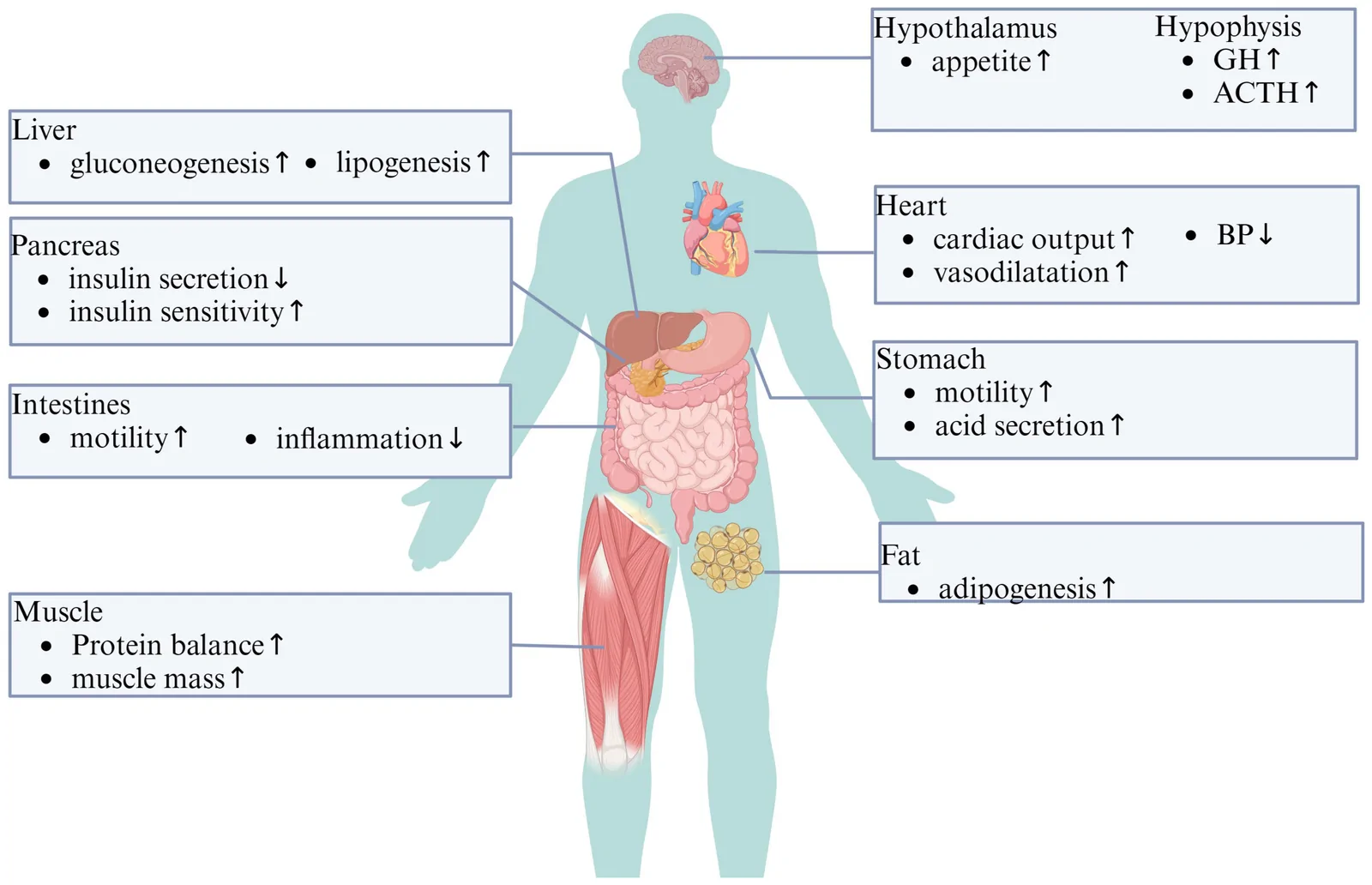
The effects of ghrelin on various organs of the body. ↑/↓, increase/decrease; GH, growth hormone; ACTH, adrenocorticotropic hormone; BP, blood pressure.

Ghrelin has been identified as a key player in numerous physiological functions affecting the entire body [1] (Figure 2). It stimulates appetite, promotes carbohydrate use as fuel while sparing fat, inhibits lipid oxidation and lipogenesis, and stimulates gastric acid secretion and motility [13]. Additionally, it impacts cardiac performance and blood pressure regulation, and has protective effects on the kidneys, heart, and brain [14]. Its involvement in cognitive functions such as learning, memory, cognition, and reward, as well as its effects on sleep, taste sensation, olfaction, and sniffing, further underscores its wide-ranging impact [15]. Ghrelin also influences pain response, microbial activity, fibrosis, bone formation, muscle excitability and regeneration, puberty, fetal lung development, hormone secretion, insulin release, and wound healing [1]. This comprehensive range of effects suggests the potential for ghrelin in therapeutic applications across various systems.

The physiological effects of ghrelin on the GI system are extensive and multifaceted, impacting a range of processes from appetite regulation to gastric motility and energy homeostasis [13,16,17,18]. This peptide hormone is predominantly produced in the stomach and exerts its effects through various mechanisms, including the hypothalamic neuropeptide Y (NPY) and Y (1) receptors, which stimulate the vagus nerve to promote appetite and GI motility [19,20]. Ghrelin secretion increases during fasting, stimulating hunger, and decreases after eating, the effect of which is more prominent in females [16]. Besides regulating appetite, ghrelin contributes to GI motility by interacting with other GI hormones such as cholecystokinin(CCK) and 5-hydroxytryptamine [21]. It enhances peristalsis and phasic contractions in the intestinal tube and colorectum, thereby facilitating GI motility [21]. Ghrelin also influences gastric acid secretion, evidenced by the intracerebroventricular injection of ghrelin promoting secretion via the vagus nerve [22]. The nitric oxide (NO) pathway likewise enhances the secretion of gastric acid and the motility of the stomach in response to ghrelin [23]. Injecting ghrelin into the duodenum enhances pancreatic enzyme secretion through vagus stimulation and CCK release [24]. In rat models of diabetes, ghrelin distribution is altered, suggesting a potential relationship with insulin secretion [25]. These findings highlight the multi-functional nature of ghrelin and its potential roles in various physiological and pathology processes related to the GI tract. Several ghrelin analogues, such as EXT418 and anamorelin, have shown promising therapeutic effects in cancer-associated cachexia [26,27].

## 3. The Mechanisms Underlying the Therapeutic Effects of Ghrelin in GI Diseases

### 3.1. Ghrelin and Autophagy

Autophagy plays a vital role in the GI tract, impacting numerous diseases including motility disorders, infectious and non-infectious inflammations (e.g., Helicobacter pylori infection, chronic gastritis, and Crohn’s disease), and GI cancers. It is crucial for maintaining intestinal epithelial homeostasis and integrity, regulating responses to infections like H. pylori, and influencing the development of GI cancers [28]. Emerging evidence has highlighted the profound effects of ghrelin, a gut hormone involved in energy homeostasis, in modulating this pathway in multiple diseases [29,30]. Recent studies reveal that ghrelin enhances autophagy through the activation of AMP-activated protein kinase, impacting lipid and glucose metabolism, intestinal mucosa remodeling, cardiac ischemia protection, and brain functions like learning and memory [31]. In inflammatory states, ghrelin acts as an anti-inflammatory factor by reducing autophagic flux to prevent cell injury [32]. However, the impact of ghrelin on autophagy has also been the subject of considerable debate. In chronic obstructive pulmonary disease (COPD), ghrelin has been found to inhibit autophagy in bronchial epithelial cells by suppressing the nuclear factor kappa-B (NF-κB) and protein-1 pathways, thereby attenuating disease progression and improving lung function [33]. Conversely, in myocardial hypertrophy, ghrelin has been shown to significantly enhance autophagy marker expression in myocardial cells [34]. In the context of GI diseases, ghrelin demonstrates a notable protective role by promoting autophagy. In vitro experiments have shown ghrelin’s ability to induce the apoptosis of colorectal adenocarcinoma cells through the activation of autophagy [35]. In a rat model of sepsis, ghrelin administration was found to significantly upregulate the autophagy markers LC3, ATG7, and beclin-1 in small intestinal epithelial cells during early sepsis [36]. Moreover, in a rat model of intestinal ischemia–reperfusion injury, ghrelin has been shown to activate autophagy in intestinal intraepithelial lymphocytes by modulating the NOD2/beclin-1 pathway, thereby ameliorating the GI mucosal injury caused by ischemia–reperfusion [37]. In summary, the modulation of autophagy by ghrelin in GI diseases is complex and context-dependent. It represents a promising therapeutic target for developing treatments for various disorders, especially where autophagy is impaired. Thus, understanding ghrelin’s role in autophagy provides potential therapeutic targets for various GI diseases.

### 3.2. Ghrelin and Apoptosis

Apoptosis, a critical gene-regulated process of cell death, plays a vital role in growth and development [38]. The influence of ghrelin on apoptosis varies across different cell types, notably impacting GI cells. While in diabetes-related cataracts, ghrelin inhibits lens cell apoptosis by modulating the expression of BAX and BCL-2 genes [39], it induces apoptosis in rheumatoid arthritis by decreasing the expression of precaspase-3, -8, and -9 [40]. The effect of ghrelin on GI cells, however, is more complex and debated. Some studies report no significant impact of ghrelin on apoptosis in OE-19 cells [41], whereas others demonstrate its role in reducing apoptosis in gastric mucosal cells, particularly under oxidative stress, and in promoting gastric mucosal repair [42]. Additionally, ghrelin inhibits apoptosis in gastric mucosal cells infected by Helicobacter pylori, achieved through upregulating constitutive NO synthase activity via the Src/Akt signal pathway, leading to reduced iNOS activity and NO production [43]. Furthermore, ghrelin’s antiapoptotic effect in GI diseases involves activating the PI3K/Akt pathway through miR-21 regulation and modulating BCL-2, BAX, and caspase-3 expression via the unfolded protein response pathway [44,45]. In most cases, ghrelin inhibits apoptosis, providing protection and facilitating the repair of damaged cells, thus exhibiting a protective role in the biological system. Under these conditions, ghrelin can exert a therapeutic effect. However, in tumor cells, ghrelin’s inhibition of apoptosis may increase tumor proliferation and lead to drug resistance, making it necessary to consider ghrelin as a potential target for therapy. Therefore, the impact of ghrelin on different cell types holds varied potential significance for the future and warrants further exploration.

### 3.3. Ghrelin and the Inflammatory Response

Inflammatory responses are significantly associated with a variety of GI diseases. Ghrelin exhibits an anti-inflammatory role in various inflammation-related diseases, such as sepsis-associated acute respiratory distress syndrome [46], colitis [47], and rheumatoid arthritis [48]. A key mechanism through which ghrelin exerts its anti-inflammatory effects is by significantly inhibiting the release of pro-inflammatory factors. For instance, in diabetes-associated lung inflammation, exogenous ghrelin has been found to substantially decrease the release of pro-inflammatory mediators TNF-α and IL-1β in the lungs [49].

The anti-inflammatory response to ghrelin involves an intricate interplay of various pathways. Ghrelin effectively suppresses the activation of the NF-κB signal pathway, thereby exerting control over downstream inflammatory mediator production and expression [50,51]. In a mouse model of liver ischemia–reperfusion injury, ghrelin demonstrates a remarkable ability to reduce the release of the pro-inflammatory factor myeloperoxidase by blocking the ERK signal pathway [52]. Ghrelin also exhibits inhibitory effects on the release of certain pro-inflammatory cytokines, such as high-mobility-group protein 1 (HMGB1), during advanced stages of inflammation [53]. In addition, through the activation of the central vagus nerve, it stimulates IL-10, an anti-inflammatory cytokine, ultimately resulting in a reduction in pro-inflammatory cytokines like IL-1a, IL-1b, and TNF-a [54]. Given ghrelin’s anti-inflammatory properties, it may possess significant therapeutic potential in treating inflammation-related disorders such as acute respiratory distress syndrome, colitis, and sepsis.

## 4. Ghrelin and GI Disorders

**Figure 3:**
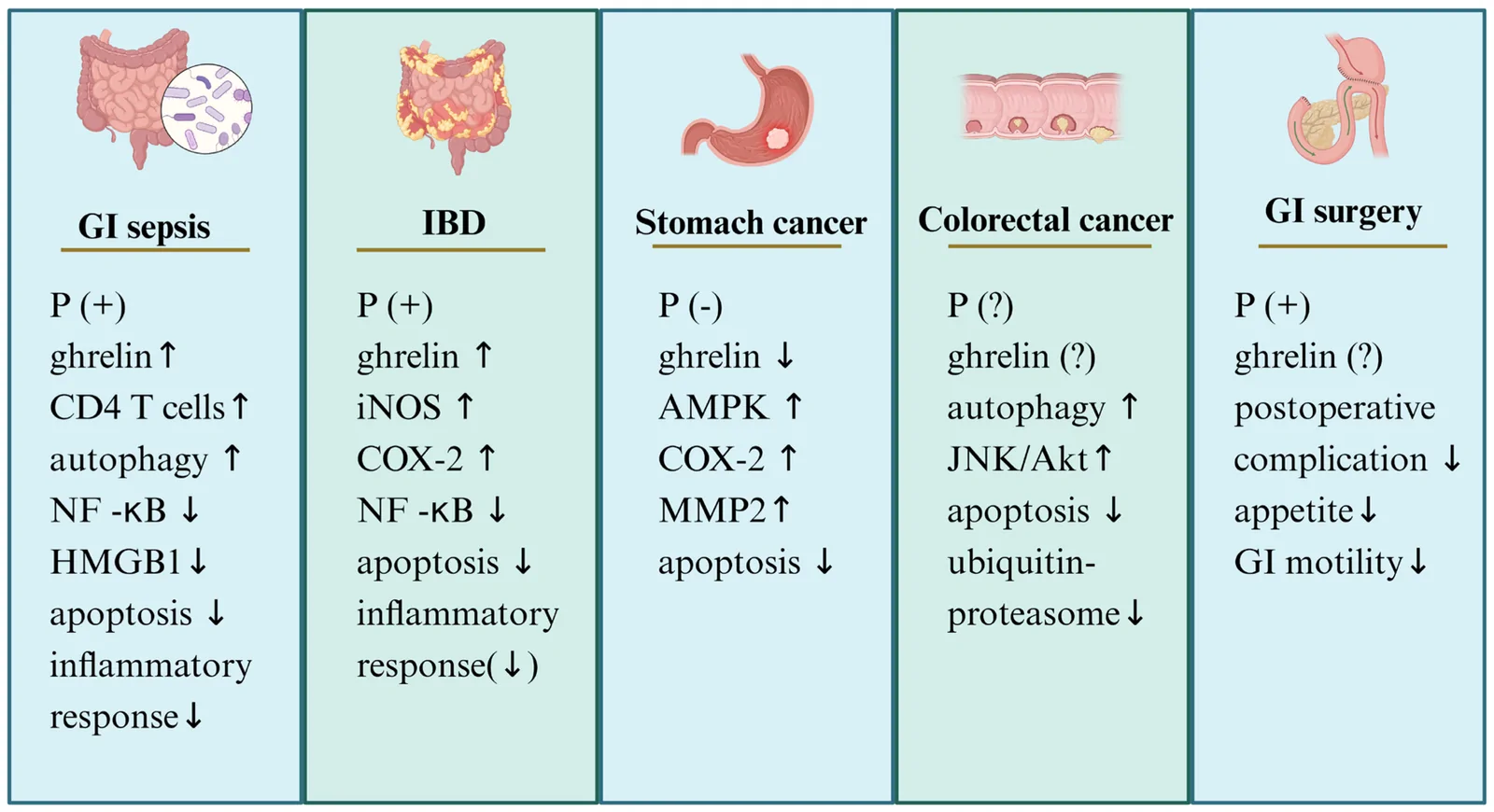
Summary diagram of the effects of ghrelin in treating gastrointestinal (GI) diseases. In GI sepsis, ghrelin can alleviate damage by inhibiting inflammatory responses, increasing GI blood flow, enhancing autophagy, and reducing cell apoptosis. In inflammatory bowel disease (IBD), serum ghrelin levels serve as markers to differentiate between active and remission phases, and ghrelin also exhibits significant anti-inflammatory effects. In gastric cancer, ghrelin acts as an early risk marker, and, due to its crucial role in promoting gastric cancer cell proliferation and migration, the ghrelin–GHS-R axis holds promise as a target for gastric cancer treatment. The role of ghrelin in colorectal cancer (CRC) remains controversial; however, ghrelin analogs have shown substantial benefits in treating cancer cachexia associated with CRC. Post-GI surgery, ghrelin or its analogs can aid in enhancing recovery and reducing complications. ↑/↓, increase/decrease; HMGB1, high-mobility-group proteins; AMPK, AMP-activated protein kinase; MMP2, matrix metalloproteinase 2; iNOS, inducible nitric oxide synthase; COX-2, cyclooxygenase-2; P, therapeutic effect; (?), controversial; (+), beneficial; (-), detrimental.

While ghrelin is widely recognized for its role in appetite regulation, it exhibits a wide range of further regulatory functions. Figure 3 presents an overview of ghrelin’s therapeutic effects in GI-related conditions including sepsis, GI surgery, IBD, gastric cancer, and colon cancer.

### 4.1. Ghrelin and GI Sepsis

In the context of sepsis, ghrelin engages directly with GHS-R, orchestrating a cascade of beneficial effects [55]. These include dampening inflammatory responses, augmenting tissue perfusion, promoting autophagy in intestinal epithelial cells, curtailing cell apoptosis, and mitigating catabolic metabolism, collectively contributing to the safeguarding of GI function. Notably, ghrelin ranks as one of the earliest hormones to surge during the sepsis course [56,57]. Research underscores that elevated plasma ghrelin levels in sepsis patients correlate with an enhanced prognosis and abbreviated ICU stays [57,58], signifying its prognostic relevance. Furthermore, an upsurge in plasma ghrelin expression has been observed to markedly ameliorate acute GI mucosal injuries resulting from sepsis [59].

The mechanistic pathways underlying ghrelin’s GI protective role are multifaceted (refer to Table 1). Primarily, ghrelin mediates anti-inflammatory responses by inhibiting oxidative stress and ameliorating GI dysfunctions induced by sepsis. Intriguingly, in the latter stages of sepsis, it also attenuates the levels and release of the pro-inflammatory factor HMGB1 [60,61]. Additionally, by activating the vagus nerve and modulating plasma HMGB1, ghrelin enhances GI mucosal functionality, thus reducing bacterial translocation and mucosal permeability [62]. Further, through the upregulation of transcription factor E2F1 and suppression of the NF-κB signaling pathway, ghrelin exerts its anti-inflammatory influence [63].

Recent advancements in sepsis research, particularly within a rat model, reveal that ghrelin’s capacity to inhibit the release of inflammatory cytokines is cholinergically mediated. This pathway promotes the transcription and translation of peptide transporter 1 (PepT1), culminating in enhanced gastric blood perfusion during septic episodes [64,65]. Finally, recent studies in a sepsis rat model have further shown that ghrelin’s inhibitory effects on inflammatory cytokine release are mediated through cholinergic neurons in a process that promotes the transcription and translation of PepT1 and leads to increased gastric blood perfusion during sepsis [66,67].

In conclusion, ghrelin’s varied actions during sepsis highlight its potential as a valuable therapeutic agent. By modulating critical pathophysiological processes, it stands as a promising candidate for enhancing the treatment of GI sepsis. Continued research into ghrelin’s mechanisms is vital for advancing our understanding and management of this challenging condition.

**Table 1:** Mechanisms by which ghrelin reduces GI damage from sepsis.

Main Effect	Mechanism	Ref.
Tissue perfusion ↑	Downregulates expression of ET-1 by regulating the NF-κB pathway.	[68]
Inflammatory response ↓	Inhibits sympathetic nerve activity through central nervous system GHS-R.	[69]
Inflammatory response ↓	Inhibits release of HMGB1 by macrophages at the end of sepsis.	[61]
Intestinal barrier function ↑	Activates the vagus nerve through central nervous system GHS-R, which ultimately reduces serum HMGB1.	[62]
Autophagy ↑	Enhances the autophagy of small intestinal epithelial cells.	[36]
Intestinal absorption ↑	1. Reduces the inflammatory response of intestinal epithelial cells. 2. Increases the expression of PepT1.	[70]
Immunity ↑	Increases proliferation of CD4 T cells.	[71]
Apoptosis ↓	Inhibits apoptosis of gastric epithelial cells.	[65]
Inflammatory response ↓	Promotes intestinal sepsis by increasing E2F1 and inhibiting the NF-κB pathway.	[63]
Intestinal absorption ↑	Inhibits inflammatory cytokine release by stimulating cholinergic neurons and thus promoting the transcription and translation of PepT1.	[64]

↑/↓, increase/decrease; HMGB1, high-mobility-group protein 1.

### 4.2. Ghrelin and Inflammatory Bowel Disease 

Alterations in serum ghrelin levels among inflammatory bowel disease (IBD) patients have catalyzed research into its potential as a biomarker for this ailment. A recent meta-analysis brought to light that, in comparison to their levels in healthy individuals, ghrelin levels significantly escalate during the active phases of IBD, yet show no substantial variance during remission periods. Moreover, a notable reduction in the obestatin/ghrelin ratio is observed during IBD’s active phases. Consequently, these findings posit both ghrelin levels and the obestatin/ghrelin ratio as potential indicators for differentiating between active and remission states of IBD [72].

Although the aforementioned changes in ghrelin levels are well documented, their impact on colitis continues to be a topic of contention. Several early studies and analyses have proposed that ghrelin might amplify the severity of IBD. Specifically, exogenous ghrelin is demonstrated to elevate neutrophil infiltration and colonic IL-1β levels, thereby exacerbating the inflammatory response in IBD [73,74]. In addition, ghrelin is known to activate macrophages via growth hormone-releasing peptides, further contributing to inflammation [75].

Conversely, a significant body of recent research and scholarly opinion suggests that ghrelin exhibits a broad spectrum of anti-inflammatory actions in IBD. It plays a crucial role in reducing cell apoptosis, enhancing colonic blood perfusion and preserving the integrity of the intestinal barrier. The therapeutic potential of ghrelin in IBD is possibly linked to the activation of the vagus nerve [76] and the transcriptional activity of peroxisome proliferator-activated receptor gamma (PPARγ) [77]. For instance, in an acetic acid-induced colitis rat model, ghrelin was shown to downregulate key inflammatory factors, such as IL-1β, TNF-α, and myeloperoxidase, providing a protective effect [78]. In the context of a trinitrobenzene sulfonic acid-induced colitis rat model, the administration of exogenous ghrelin was observed to facilitate the release of NO and prostaglandin E2 by augmenting iNOS and COX-2 expression. This process stimulates the release of sensory neuropeptides, like calcitonin gene-related peptide (CGRP), from sensory nerve terminals, which aids in the healing of colonic lesions [79]. Furthermore, in a mouse model of trinitrobenzene sulfonic acid-induced colitis, ghrelin effectively downregulated Th1 cell-induced autoimmune responses and the production of inflammatory mediators, thereby safeguarding the GI barrier, mitigating weight loss, reducing diarrhea, decreasing mortality rates, and preventing disease recurrence [80]. In a dextran sulfate sodium (DSS)-induced mouse model of colitis, ghrelin was shown to alleviate intestinal barrier dysfunction by suppressing the NF-κB pathway, which in turn mitigated inflammation [81]. Additionally, ghrelin contributes to reducing apoptosis in colonic cells by diminishing the expression of pro-apoptotic markers like Bax, Gpr78, and C/EBP homologous protein (Chop) mRNA, while concurrently increasing the expression of the anti-apoptotic protein Bcl-2 [45]. In another acetic acid-induced colitis rat model, ghrelin markedly improved colonic blood perfusion, enhanced superoxide dismutase (SOD) activity, and lowered the concentrations of IL-1β and MDA, thus exerting a protective role [82]. Lastly, studies in aged GHS-R knockout mice (*Ghsr−/−*) and ghrelin knockout mice (*Ghrl−/−*) have revealed alterations in the intestinal microbiome and in tryptophan metabolism, respectively, leading to increased susceptibility to IBD [47,83], further substantiating the protective role of ghrelin.

In light of these varied findings, ghrelin emerges as a multifaceted molecule with significant implications for IBD treatment due to its ability to suppress inflammatory responses and its impact on the physiology and pathology of the intestines. This positions it as a promising candidate for novel IBD treatment strategies. Ongoing research efforts, aimed at unraveling the complex mechanisms of ghrelin, are paving the way for more effective and targeted therapeutic approaches, bringing hope for improved management and prognosis in IBD patients.

### 4.3. Ghrelin and Gastric Cancer

Several studies have explored the relationship between circulating ghrelin levels and the risk of gastric cancer, indicating that serum ghrelin levels can serve as an early biomarker for the risk of gastric cancer [84,85]. A recent meta-analysis has shown that patients with gastric cancer have significantly lower circulating ghrelin levels compared to those of healthy individuals [86]. Moreover, plasma ghrelin levels are negatively correlated with the degree of gastric mucosal atrophy and intestinal metaplasia, and can predict the risk of developing gastric cancer, regardless of H. pylori infection [87]. Mansour-Ghanaei proposed that serum levels of pepsinogen I and the pepsinogen I/II ratio could be combined with ghrelin to act as biomarkers for gastric cancer screening [88].

Compared to that in normal tissues, the expression level of ghrelin in the tumor tissues of gastric cancer patients is significantly lower [89,90]. Studies have also observed that the expression of ghrelin in gastric adenocarcinoma cells is almost negligible [91]. However, it is noteworthy that studies indicate a significant correlation where patients with high ghrelin expression in cancer cells tend to have a higher tumor stage and shorter survival times for gastric cancer [89,92].

Ghrelin can promote the proliferation and migration of gastric cancer cells through various mechanisms, including inhibiting apoptosis, altering the immune microenvironment, and acting through multiple signaling pathways. In vitro experiments have shown that applying exogenous ghrelin to gastric cancer cells increases COX-2 expression via the PI3K/Akt pathway, thereby inhibiting apoptosis and promoting proliferation and metastasis [93]. Another study indicates that the ghrelin/GHS-R signaling pathway can suppress the expression of the tumor suppressor gene *p53* and promote the expression of the oncogene *CDK6* [94]. Additionally, ghrelin can upregulate the expression of the metastatic factor MMP2 through the NF-κB pathway, thus facilitating the migration and invasion of gastric cancer cells [94]. Bioinformatic analysis has shown that ghrelin can alter the infiltration levels of immune cells such as B cells, CD8+ T cells, and CD4+ T cells, and macrophages in gastric cancer tissues, thereby modulating the tumor’s immune microenvironment and promoting the progression of gastric cancer cells [89].

Therefore, in the future, ghrelin and GHS-R may become potential therapeutic targets for gastric cancer treatment.

### 4.4. Ghrelin and Colorectal Cancer

The association between serum ghrelin and colorectal carcinoma (CRC) remains a subject of controversy. Some studies have indicated markedly reduced serum ghrelin levels in individuals with CRC compared to those in their healthy counterparts, with variations based on tumor location, H. pylori infection, and tumor stage [95,96]; others have observed higher serum ghrelin levels in CRC patients [97]. However, it has also been demonstrated that ghrelin levels do not substantially change during the development of CRC [98], suggesting that ghrelin may not serve as an early predictor of CRC. It is noteworthy that the latest genome-wide association studies (GWAS) and Mendelian randomization (MR) analyses suggest a potential causal relationship between elevated plasma ghrelin levels and a reduced risk of GI cancer [99]. Additionally, recent studies have identified significantly higher expression of ghrelin and GHS-R in colorectal adenomas compared to that in surrounding tissues [100]. All told, further research is warranted to explore plasma ghrelin levels in the CRC population, along with their relationship to factors such as patient heterogeneity and tumor staging [101].

Interestingly, genetic studies have revealed the *GHS-R* gene to exhibit significant hypermethylation in CRC tissues compared to that in normal mucosa, while ghrelin gene methylation is not significantly changed [102]. Furthermore, hypermethylation of *GHS-R* is present regardless of whether the adenoma is in the early or advanced stage [102].

The influence of ghrelin on CRC remains a topic of considerable debate. Some scholars advocate that the ghrelin–GHS-R axis could potentially inhibit the onset and progression of CRC. In vitro studies have revealed that introducing exogenous ghrelin can lead to a reduction in ubiquitin–proteasome activity and an enhancement of autophagy, thereby promoting apoptosis in CRC cells [35]. In an inflammation-related colon cancer model induced by azoxymethane (AOM)/dextran sulfate sodium (DSS), it was observed that *Ghrl(−/−)* mice developed larger colon tumors [103]. Correspondingly, in vivo experiments with intestinal carcinogenesis mouse models have demonstrated that ghrelin might decrease the occurrence of inflammation-related colon cancer [103]. Recent research further highlights a correlation between low plasma ghrelin levels, obesity, insulin resistance, and increased vulnerability to colon cancer [104].

Conversely, another group of scholars argues that ghrelin could potentially accelerate the invasiveness of CRC [105,106]. Studies have shown that the external addition of ghrelin increases proliferation and invasion in SW480 cells [105]. Notably, in CRC xenograft mouse models, GHS-R1α knockout resulted in a significant reduction in xenograft tumor weights [105]. Further in vitro research indicates that ghrelin-induced proliferation in CRC cells is associated with the activation of the Ras/PI3K/Akt/mTOR pathway [106]. Additionally, investigations into the Caco-2 cell line suggest that ghrelin can facilitate the transition of cells from the G1 phase to the S phase through the phosphorylation of PI3K-Akt [107].

A particularly severe complication in the terminal stages of CRC is cancer cachexia, which drastically impairs patients’ quality of life. Research has pointed to a correlation between ghrelin deficiency and CRC cachexia, suggesting the viability of exogenous ghrelin treatment for this condition [98]. The administration of the ghrelin analog GHPR-2 has been shown to improve appetite and body weight in tumor-bearing mice with early-stage anorexia/cachexia syndrome [108]. In co-culture systems involving CT26 colon cancer and C2C12 myoblast cell lines, ghrelin has been proven to inhibit apoptosis in C2C12 cells. This effect is primarily achieved by activating the JNK/Akt pathway, decreasing pro-apoptotic BAX expression, and increasing anti-apoptotic BCL-2 expression [109]. Moreover, ghrelin has been found to inhibit muscle-specific calpain activity in CT26 type CRC mice, effectively improving their muscle mass and nutritional status. Anamorelin, an orally administered ghrelin analog binding to GHS-R, has been approved in Japan for treating cancer cachexia characterized by weight loss and anorexia. Clinical trials in phases II and III have observed improvements in patient weight, appetite, and grip strength, with no significant safety risks [27,110,111].

In summary, there is a clear necessity for more comprehensive studies to unravel the contradictory roles of ghrelin in CRC. Future research should aim to delineate the precise molecular pathways through which ghrelin influences CRC development, considering the potential therapeutic implications of modulating ghrelin or GHS-R activity. The effectiveness of ghrelin analogs like anamorelin in treating cancer cachexia, as evidenced in clinical trials, demonstrates the potential of ghrelin-targeted therapies. However, the intricate balance between ghrelin’s protective and potentially tumorigenic effects calls for a cautious and nuanced approach in leveraging its therapeutic potential. Ultimately, continued research into ghrelin’s multifaceted role in CRC will be crucial in developing novel strategies for cancer prevention, diagnosis, and treatment, potentially transforming the clinical management of CRC.

### 4.5. Ghrelin and GI Surgery

After GI surgery, there is a significant decrease in ghrelin levels. Depending on the purpose of the surgery, this can have both positive and negative effects [26,112]. A reduction in ghrelin can lead to weight loss and poor appetite in some patients [113], which may be beneficial for those undergoing weight loss surgery [112]; however, it can also contribute to malnutrition and poor healing post-surgery, particularly in patients experiencing difficulties in eating, nausea, and vomiting, as well as in certain cancer patients [26,114]. Several factors contribute to this decrease, including the removal of or an alteration in the stomach and/or intestines during surgery, leading to a reduced population of cells capable of producing ghrelin and subsequent changes in plasma ghrelin concentration [115]. Changes in food digestion and absorption after surgery can also affect ghrelin secretion [116], while postoperative pain, stress, and inflammation may contribute to decreased ghrelin levels [117,118]. Notably, after Roux-en-Y gastric bypass (RYGB) bariatric surgery, increased ghrelin production is detected in the stomach, but no significant alteration in serum ghrelin is observed [119]; meanwhile, after single gastric bypass (OAGB), serum ghrelin secretion shows no significant difference [120].

The relationship between ghrelin and surgical complications has been explored, with reports that low plasma ghrelin on the first postoperative day is associated with postoperative complications [121] and that a lower ratio of preoperative plasma AG to DAG is associated with a higher probability of postoperative complications [122]. These findings suggest that managing ghrelin levels before and after surgery may serve as a preventive measure against certain serious surgical complications. Accordingly, extensive research has been conducted to explore the therapeutic benefits of ghrelin after GI surgery. The results show that ghrelin treatment following gastrectomy can improve eating patterns and effectively address decreased appetite and weight loss [123], and that elevated post-surgery plasma ghrelin promotes GI activity and accelerates gastric emptying [124]. Such acceleration can shorten the time to occurrence of first bowel sounds and movements in patients after surgery [125], while also reducing peritoneal adhesion formation and fibrotic response [126]. Ghrelin may also exhibit a potential therapeutic effect on postoperative intestinal obstruction, as the downregulation of GHS-R in the small intestinal muscle layer may be associated with the development of postoperative small bowel dyskinesia [127].

In summary, ghrelin treatment can improve dietary patterns and counteract the decrease in appetite and weight loss after gastrectomy. Additionally, elevated post-surgery plasma ghrelin levels may promote gastrointestinal activity, accelerate gastric emptying, and potentially reduce complications such as peritoneal adhesion and postoperative intestinal obstruction. These promising results suggest that ghrelin or its analogs could be key in enhancing postoperative recovery and reducing complications.

## 5. Conclusions

This review of ghrelin’s role in the GI system underscores its significance in a range of disorders. Its involvement in autophagy, apoptosis, and inflammatory responses demonstrates its potential as a therapeutic target. The complex relationship between ghrelin levels and various GI disorders, including IBD, gastric and colorectal cancers, and post-surgical recovery, reveals a hormone with multifaceted impacts on GI health. This research highlights the necessity for the continued exploration of ghrelin’s mechanisms of action and therapeutic applications, offering promising avenues for future medical advancements in treating GI diseases. In conclusion, ghrelin, as a multifunctional peptide hormone, emerges as a pivotal player in the GI system with broad implications for various GI diseases and disorders. Its influence on cellular pathways, inflammation, cell proliferation, appetite regulation, and GI motility, along with its roles in postoperative complications and its potential as a therapeutic target, underscore the need for deeper understanding and research to fully harness its therapeutic capabilities in managing GI diseases and disorders.

## Data Availability

No new data were created or analyzed in this study.
