# Correction: Wu et al. Ghrelin in Focus: Dissecting Its Critical Roles in Gastrointestinal Pathologies and Therapies. *Curr. Issues Mol. Biol.* 2024, *46*, 948–964

**DOI:** 10.3390/cimb48111151

**Published:** 2026-11-03

**Authors:** Wei Wu, Lei Zhu, Zhimin Dou, Qiliang Hou, Sen Wang, Ziqian Yuan, Bin Li

**Affiliations:** 1 Department of Intensive Care Medicine, The First School of Clinical Medicine, Lanzhou University, Lanzhou 730000, China; wuw21@lzu.edu.cn (W.W.); qilianghou@outlook.com (Q.H.); 220220909171@lzu.edu.cn (S.W.); yuanzq331@163.com (Z.Y.); 2 Department of Intensive Care Medicine, The First Hospital of Lanzhou University, Lanzhou 730000, China; zhulei0224@163.com (L.Z.); douzhm@126.com (Z.D.)

**Text Correction**

In the original publication [1], since the paper “Ghrelin Alleviates Intestinal Dysfunction in Sepsis Through the KLF4/MMP2 Regulatory Axis by Activating SIRT1” cited in this review has been withdrawn, all references to this cited paper and the sections related to it have been removed. In the second paragraph of Section 4.1, “Ghrelin and GI Sepsis”, the following content has been deleted: “Moreover, ghrelin’s activation of sirtuin 1 (SIRT1) expression, leading to the upregulation of Kruppel-like factor 4 (KLF4) and subsequent downregulation of matrix metalloproteinase-2 (MMP2), plays a critical role in lessening GI injury [64].”

**Error in Table**

Since the paper “Ghrelin Alleviates Intestinal Dysfunction in Sepsis Through the KLF4/MMP2 Regulatory Axis by Activating SIRT1” cited in this review has been withdrawn, the following content in the tenth row of Table 1 has been deleted: “Inflammatory response ↓ Inhibits inflammation by activating SIRT1 and modulating the KLF4/MMP2 regulatory axis [64]”. The correct legend appears below.

All instances of reference [64] have been deleted, and the order of some references has been adjusted accordingly.

The authors state that the scientific conclusions are unaffected. This correction was approved by the Academic Editor. The original publication has also been updated.

**Table 1:** Mechanisms by which ghrelin reduces GI damage from sepsis.

**Main Effect**	**Mechanism**	**Ref.**
Tissue perfusion ↑	Downregulates expression of ET-1 by regulating the NF-κB pathway.	[68]
Inflammatory response ↓	Inhibits sympathetic nerve activity through central nervous system GHS-R.	[69]
Inflammatory response ↓	Inhibits release of HMGB1 by macrophages at the end of sepsis.	[61]
Intestinal barrier function ↑	Activates the vagus nerve through central nervous system GHS-R, which ultimately reduces serum HMGB1.	[62]
Autophagy ↑	Enhances the autophagy of small intestinal epithelial cells.	[36]
Intestinal absorption ↑	1. Reduces the inflammatory response of intestinal epithelial cells. 2. Increases the expression of PepT1.	[70]
Immunity ↑	Increases proliferation of CD4 T cells.	[71]
Apoptosis ↓	Inhibits apoptosis of gastric epithelial cells.	[65]
Inflammatory response ↓	Promotes intestinal sepsis by increasing E2F1 and inhibiting the NF-κB pathway.	[63]
Intestinal absorption ↑	Inhibits inflammatory cytokine release by stimulating cholinergic neurons and thus promoting the transcription and translation of PepT1.	[64]

↑/↓, increase/decrease; HMGB1, high-mobility-group protein 1.

## References

[1] Wu W., Zhu L., Dou Z., Hou Q., Wang S., Yuan Z., Li B. (2024). Ghrelin in Focus: Dissecting Its Critical Roles in Gastrointestinal Pathologies and Therapies. Curr. Issues Mol. Biol..

